# Declining trend of *Plasmodium falciparum dihydrofolate reductase (dhfr) and dihydropteroate synthase (dhps)* mutant alleles after the withdrawal of Sulfadoxine-Pyrimethamine in North Western Ethiopia

**DOI:** 10.1371/journal.pone.0126943

**Published:** 2015-10-02

**Authors:** Sofonias K. Tessema, Moges Kassa, Amha Kebede, Hussein Mohammed, Gemechu Tadesse Leta, Adugna Woyessa, Geremew Tasew Guma, Beyene Petros

**Affiliations:** 1 Department of Biology, Faculty of Science, Addis Ababa University, Addis Ababa, Ethiopia; 2 Ethiopian Public Health Institute, Addis Ababa, Ethiopia; Université Pierre et Marie Curie, FRANCE

## Abstract

Antimalarial drug resistance is one of the major challenges in global efforts of malaria control and elimination. In 1998, chloroquine was abandoned and replaced with sulfadoxine/pyrimethamine, which in turn was replaced with artemether/lumefantrine for the treatment of uncomplicated *falciparum* malaria in 2004. Sulfadoxine/pyrimethamine resistance is associated with mutations in dihydrofolate reductase (*Pfdhfr*) and dihydropteroate synthase (*Pfdhps*) genes. The prevalence of mutation in *Pfdhfr* and *Pfdhps* genes were evaluated and compared for a total of 159 isolates collected in two different time points, 2005 and 2007/08, from Pawe hospital, in North Western Ethiopia. The frequency of triple *Pfdhfr* mutation decreased significantly from 50.8% (32/63) to 15.9% (10/63) (*P*<0.001), while *Pfdhps* double mutation remained high and changed only marginally from 69.2% (45/65) to 55.4% (40/65) (*P* = 0.08). The combined *Pfdhfr/Pfdhps* quintuple mutation, which is strongly associated with sulfadoxine/pyrimethamine resistance, was significantly decreased from 40.7% (24/59) to 13.6% (8/59) (*P*<0.0001). On the whole, significant decline in mutant alleles and re-emergence of wild type alleles were observed. The change in the frequency is explained by the reduction of residual drug-resistant parasites caused by the strong drug pressure imposed when sulfadoxine/pyrimethamine was the first-line drug, followed by lower fitness of these resistant parasites in the absence of drug pressure. Despite the decrease in the frequency of mutant alleles, higher percentages of mutation remain prevalent in the study area in 2007/08 in both *Pfdhfr* and *Pfdhps* genes. Therefore, further multi-centered studies in different parts of the country will be required to assess the re-emergence of sulfadoxine/pyrimethamine sensitive parasites and to monitor and prevent the establishment of multi drug resistant parasites in this region.

## Introduction

For more than 40 years, chloroquine was the first line anti-malarial drug in Ethiopia for the treatment of uncomplicated *Plasmodium falciparum* malaria. In 1998, chloroquine (CQ) was abandoned and replaced with sulfadoxine/pyrimethamine (SP). In 2004, SP was replaced with artemether/lumefantrine (AL) for the treatment of uncomplicated *falciparum* malaria [1,2]. SP is no longer recommended for the treatment of malaria but it remains a vital tool to reduce the burden of malaria in Africa. The World Health Organization (WHO) recommends SP for intermittent preventive treatment in pregnancy (IPTp), in infancy (IPTi) and in childhood (IPTc) [3–5]. In sub-Saharan Africa, 39 countries had adopted intermittent preventive treatment in pregnancy between 1993 and 2007 [6].

Point mutations in *P*. *falciparum dihydrofolate reductase* (*Pfdhfr*) and *dihydropteroate synthase* (*Pfdhps*) genes are known to confer resistance to pyrimethamine and sulfadoxine, respectively [7,8]. Pyrimethamine resistance is generally caused by three mutations in *Pfdhfr* gene, namely: N51I, C59R, and S108N [9]; sulfadoxine resistance in sub Saharan Africa is caused by substitutions S/A436F, A437G, K540E, A581G, and A613S/T in a variety of combinations in *Pfdhps* gene [10]. Parasites carrying all five mutations, *Pfdhfr* triple mutant (51I, 59R, and 108N) and *Pfdhps* double mutant (437G and 540E), commonly called quintuple mutations have been strongly associated with SP treatment failure in sub-Saharan Africa [11–13]. Additionally, *Pfdhfr* (I164L) and increased prevalence of the *Pfdhps* A581G mutation has been well documented and are linked with increased therapeutic failure of SP in southeastern Africa [14–17]. WHO recommends the implementation of SP for intermittent preventive treatment only if the prevalence of the K540E mutation (and thus the quintuple mutation) is <50% [18].

Following the withdrawal of CQ in 1993, *Pfcrt* mutation (K76T) gradually decreased and disappeared completely by 2001 in Malawi [19]. Similar findings have been reported in Tanzania [20], Kenya [21] and China [22]. This decline was correlated with the return of the clinical efficacy of CQ for the treatment of *falciparum* malaria in Malawi [23]. In Cambodia, however, alleles conferring CQ and SP resistance occur at a high frequency after the withdrawal of these drugs [24]. In Venezuela, the complete fixations of mutant *Pfdhfr* and *Pfdhps* alleles eight years after the withdrawal of SP were reported [25]. Zhou *et al*., 2009, reported decrease in the frequency of *Pfdhfr* and *Pfdhps* gene mutations after the withdrawal of SP in Peru. Decline in *Pfdhfr* and *Pfdhps* mutations after the withdrawal of SP has also been reported from Northern Ethiopia [26], Tanzania [27] and southern Mozambique [28].

In Ethiopia, high level of *Pfdhfr* triple mutations and *Pfdhfr/Pfdhps* quintuple mutations was reported from Jimma, Dilla and Bahirdar in 2004/05 [26,29,30]. A study conducted in Northern Ethiopia (Bahirdar) showed a significant decline in the triple *Pfdhfr* mutation from 78.6% in 2005 to 56.4% in 2008 and the quintuple mutations reduced from 60.6% to 37.2% between 2005 and 2008 [31]. In this study we evaluated change in the frequency of the mutant and wild type *Pfdhfr* and *Pfdhps* markers after the withdrawal of SP in 2004 in a perennial transmission setting. The objective of this study was to determine the change in the frequency of *Pfdhfr* and *Pfdhps* mutant and wild alleles in *P*. *falciparum* isolates collected in 2005 and 2007/08.

## Materials and Methods

### Study area

**Fig 1:**
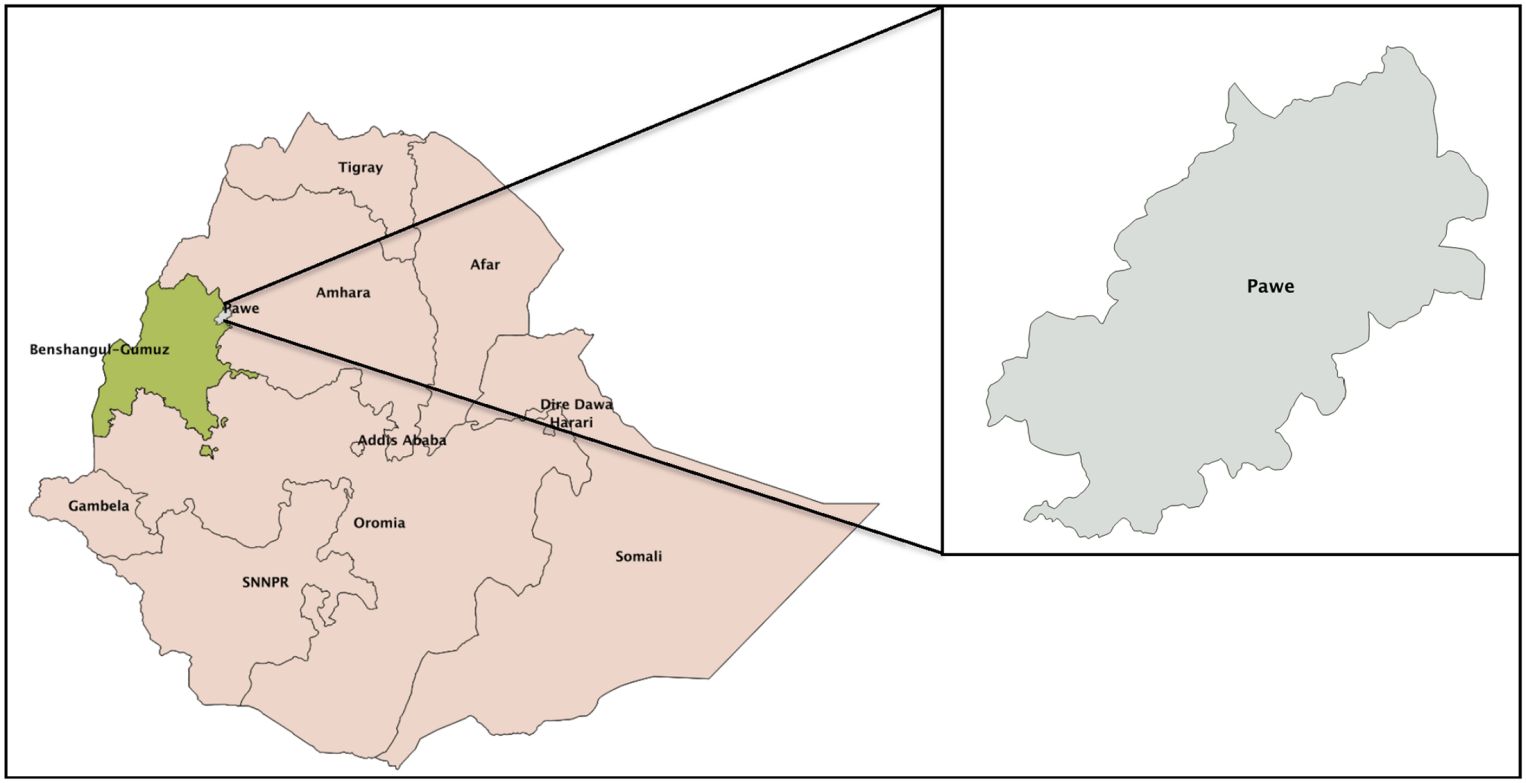
Map of the study area.

The study was conducted in the rural town of Pawe, in the North Western part of Ethiopia (Fig 1). The area is located at an altitude of 1050 meters above sea level with a mean annual temperature ranging from 16.2°C to 32.2°C, and the mean annual rainfall between 980 and 1200 mm occurring in two seasons from March to May and from June to December (Pawe Agricultural Research Centre). Pawe was one of the 14 sentinel sites, that were eco-epidemiologically selected, for drug resistance studies by the Federal Ministry of Health [2].

### Study population

*Plasmodium falciparum* samples were collected from 80 patients in 2005 by the Ethiopian Public Health Institute (formerly known as Ethiopian Health and Nutrition Research Institute (EHNRI)). In 2007/08, we screened 602 patients attending Pawe hospital and 79 of them were positive for *Plasmodium falciparum* malaria by light microscopy. We included patients of all age, both sex and only those who lived in the study area for at least two years. The same inclusion criteria were used in 2005. Patients with severe malaria [32], severe malnutrition and serious underlying diseases according to the physicians comment were excluded from the study.

A finger-prick blood sample was taken for thick and thin blood smear. An experienced microscopist examined the Giemsa-stained blood smears. If the patient was infected with *P*. *falciparum* mono-infection and fulfilled the inclusion criteria, two to three drops of blood was collected on to a whatman filter paper (Krackeler Scientific Inc., New York). The filter paper was labeled, dried and stored at -20°C. After the blood sample was collected, additional thin and thick blood smears were made labeled and transported to EHNRI for re-confirmation by a different microscopist and parasite count. From the thick smears, parasite density was determined by counting the number of asexual parasites against 200 white blood cells. The thin smear was used for species determination. The national and Addis Ababa University (Department of Biology) ethical boards independently approved this study. A written informed consent was translated to the local languages of the study area and approved by the ethical review boards. During sample collection a written informed consent was obtained from each participant and parents or guardian and appropriate drug was given free of charge for patients according to the physician subscription.

### DNA extraction and PCR amplification

Parasite genomic DNA was extracted from dried blood spot as previously described [33]. *Pfdhfr* and *Pfdhps* genes were amplified from 2–3μl of genomic DNA for the primary PCR and 2μL of the PCR product for the nested PCR. The sequence of the primers, PCR and cycling conditions are shown in S1 Table. Positive control genomic DNA from 3D7, Dd2, T994, HB3, T996, SL/D6 and IEC513/86 (S2 Table) and negative control were included in all PCR reactions. PCR products were visualized in agarose gel containing ethidium bromide. The bands were analyzed and the PCR products were used for the dot blot hybridization experiment. PCRs were repeated and optimized for the negative samples.

### Preparation of dot blots

PCR products were chemically denatured, heated and then neutralized with equal volume ammonium acetate (pH = 7). The denatured PCR product was blotted by direct application onto Gene screen nylon membranes using dot blotter (Bio-Rad, UK). The membrane was incubated for 30 minutes and neutralized by washing in saline-sodium citrate buffer (2X SSC) for 60 seconds. The membrane was immersed in 0.4M Sodium Hydroxide for 30–60 seconds to ensure complete denaturation of immobilized DNA, and then rinsed in neutralizing buffer for 30 seconds. The cross-linking between the applied DNA and the membranes was done using UV cross-linker as previously described [34]. In each blot appropriate positive controls for *Pfdhfr* and *Pfdhps* (S2 Table) and negative control were included.

### Labeling of oligonucleotide probes

Ten oligonucleotide probes were labeled with radioactive ^32^P as described in previous studies (Table 1) [34,35]. Briefly, 1μl (10pMol) of each oligonucleotide probe (MWG Biotech, Germany) mixed with 1μl Ready-To-Go T4 Polynucleotide Kinase (Amersham Pharmacia Biotech, UK) and 47μl of DNase free water. 1μl of [γ-32P] ATP (Institutes of Isotopes, Hungary) was added and incubated for 30 minutes at 37°C. The reaction was stopped by 5μl of 250mM EDTA. The unincorporated [32p]-γ-ATP was removed by re-suspending the mixture in column according to the manufacturer guidelines (Amersham Pharmacia Biotech, UK). The purified sample was stored in -20°C until further use. Strict personal and environmental safety protocols of the national radiation regulation authority were followed.

**Table 1:** List of oligonucleotide probes used for the detection of polymorphism in *Pfdhfr* and *Pfdhps* genes.

Gene	Amino acid	Probe sequences	Hybridization Temperature
*Pfdhfr*	Ser 108	5'-AACAAGCTGCGAAAGCATTCCAA-3'	54°C
	Asn 108	5'-AACAAACTGGGAAAACATTCCAA-3'	54°C
	Ile 51	5'-CCATGGAAATGTATTTCGCTAG-3'	45°C
	Asn 51	5'-CCATGGAAATGTAATTCGCTAG-3'	54°C
	Arg 59	5'-GAAATATTTTCGTGCAGTTAC-3'	48°C
	Cys 59	5'-GAAATATTTTTGTGCAGTTAC-3'	58°C
*Pfdhps*	Gly 437	5'-GAATCTTCTGGTCCTTTT-3'	43°C
	Ala 437	5'-GAATCCTCTGCTCCTTTT-3'	51°C
	Lys 540	5'-CAATGGATAAACTAACAA-3'	35°C
	Glu 540	5'-CAATGGATGAACTAACAA-3'	35°C

### Dot blot hybridization

The blot membrane was immersed in a hybridization buffer (Final concentration: 5X SSPE, 5X Denhardt’s reagent, 0.5% SDS, 59.9% DNase-free water and 0.01 mg/ml sonicated salmon sperm) (Gibco BRL, UK) and incubated for 30 minutes at the specific hybridization temperature of each probe (Table 1). The ^32^P labeled oligonucleotide probe (1μl for every 1ml of the hybridization buffer) was added and hybridized overnight in rotary rocker hybridization oven. The membrane was washed once in 2X SSC and twice in 1X SSC/0.1% SDS with 20 and 10 minutes incubation, respectively. The washing solution was poured off and disposed according to local radiation regulations. The blot was wrapped in cling film and taped into an autoradiography cassette (Amersham Biosciences, France). It was exposed to X-ray film (Amersham Bioscience, France) at –70°C for 12–24 hours. The film was developed and results were scored. Following autoradiography, probes were stripped off by two washes in 0.1M Sodium Hydroxide and rinsed in 5XSSC [34,35]. The blots were then re-hybridized with other probes as required.

### Data analysis

The data was scored for each sample as mutant, wild and mixed (mutant and wild) based on the detection of the specific probes. The scored raw data is attached in S1 Dataset. We scored double, triple and quintuple mutations based on the detection of specific alleles in the isolates. For example, if we detect three *Pfdhfr* mutations in the same isolate, we score this isolate as a triple *Pfdhfr* mutant and the same for the *Pfdhps* and the quintuple mutation. The changes in the frequencies of mutations between groups were compared using the F-test. The data was analyzed using GraphPad Prism v6.0 (GraphPad Software Inc).

## Results

### Malaria prevalence in the study area

A retrospective data was obtained from clinical records at Pawe hospital for the year 2005, 2006 and a three-month record from November 2007 to January 2008. In 2005/06 prevalence of malaria by microscopy was 23.1% (2440/10569); 19.8% (1671/8444) in 2006 and 14.78% (89/602) in the three month period from November, 2007 to January, 2008. By microscopy, *Plasmodium falciparum* is the pre-dominant species in the study area with 88.6%, 69.24% and 88.76% of all malaria cases in 2005, 2006 and 2007/08 (3 month) respectively. *P*. *vivax* prevalence was 9.4% in 2005/06, 3.17% in 2006/07 and 7.87% in 2007/08 (3 month) and the rest were infected with mixed infections (*P*. *falciparum* and *P*.*vivax*) (Table 2). The retrospective data showed that between 2005 and 2007 malaria accounted for 60% of admission and about 30% mortality in the hospital.

**Table 2:** Prevalence of malaria based on clinical records in Pawe general hospital from 2005/06 to 2007/08.

Year	Total (N)	Positive to malaria N (%)	*P*. *falciparum* N (%)	*P*.*vivax* N (%)	Mixed (Pf & Pv) N (%)
2005/06	10569	2440 (23.1)	2162(88.6)	229(9.4)	49(2)
2006/07	8444	1671 (19.8)	1157(69.24)	53(3.17)	461(27.59)
2007/08*	602	89 (14.78)	79(88.76)	7(7.87)	3(3.37)

*Only 3 months data is shown.

### Characteristics of study participants

The data presented in this study is based on 159 samples tested for the change in the frequency of *Pfdhfr* and *Pfdhps* mutant and wild type alleles for the year 2005 (n = 80) and 2007/08 (n = 79). The study participants between the two study periods are comparable in terms of age, sex and parasite density (Table 3). The parasite counts were in the range of 2200–180,000/μl (Geometric mean = 13554.28/μl) and 1,840–200,000/μl (Geometric mean = 14757.68/μl) in 2005 and 2007/08, respectively. Among these 80 and 79 microscopy positive samples, we successfully amplified 63 samples for *Pfdhfr* in 2005 and 2007/08 and 65 for *Pfdhps* gene by the nested PCR.

**Table 3:** Characteristics of the study participants.

Characteristics		2005 N (%)	2007/8 N (%)
Sex	Male	38(47.5)	40 (50.63)
	Female	42(52.5)	39(49.37)
Age (year)	Range	1–46	1/2–60
	≤5	23(28.75)	17(21.5)
	6–14	17(21.25)	28(35.4)
	≥15	40(50)	34(43.1)

### Prevalence of *Pfdhfr* and *Pfdhps* mutations

The prevalence of mutations in five codons, three for the *Pfdhfr* gene (N51I, C59R, and S108N) and two codons of the *Pfdhps* gene (A437G, and K540E) were analyzed to assess the change in the frequency from 2005 to 2007/8 after the withdrawal of SP from the study area. In 2005, 92% (58/63) isolates had mutation in the *Pfdhfr* codon 108, 82% (52/63) at codon 59 and 62% (39/63) at codon 51. About 75.4% (49/65) of the isolates had mutation in one of the *Pfdhps* codon in 2005. In 2007, 74.6% (47/63) of the isolates exhibited the *Pfdhfr* core mutation N108. In 2005, only four isolates displayed wild type alleles in *Pfdhfr* codon 108 (Table 4).

**Table 4:** Number and frequency of *Pfdhfr* and *Pfdhps* mutant and wild type alleles in 2005 and 2007/08 in Pawe, North Western, Ethiopia.

Year	Codon	N	Wild type N (%)	Mutant type N (%)	Mixed N (%)
2005	*Pfdhfr* S108**N**	63	4 (6.35)	58 (92.1)	1 (1.6)
	*Pfdhfr* C59**R**	63	8(12.7)	52 (82.5)	3 (4.8)
	*Pfdhfr* N51**I**	63	13(20.6)	39 (61.9)	11 (17.5)
	*Pfdhps* A437**G**	65	13 (20.0)	49 (75.4)	3 (75.4)
	*Pfdhps* K540**E**	65	10 (15.4)	52 (80.0)	3 (4.6)
2007/08	*Pfdhfr* S108**N**	63	16 (25.4)	47 (74.6)	0 (0)
	*Pfdhfr* C59**R**	63	26 (41.3)	35 (55.6)	2 (3.2)
	*Pfdhfr* N51**I**	63	30 (47.6)	16 (25.4)	17 (27.0)
	*Pfdhps* A437**G**	65	21 (32.3)	44 (67.7)	0 (0)
	*Pfdhps* K540**E**	65	24 (36.9)	41 (63.1)	0 (0)

### Temporal decline in *Pfdhfr* and *Pfdhps* mutations

**Fig 2:**
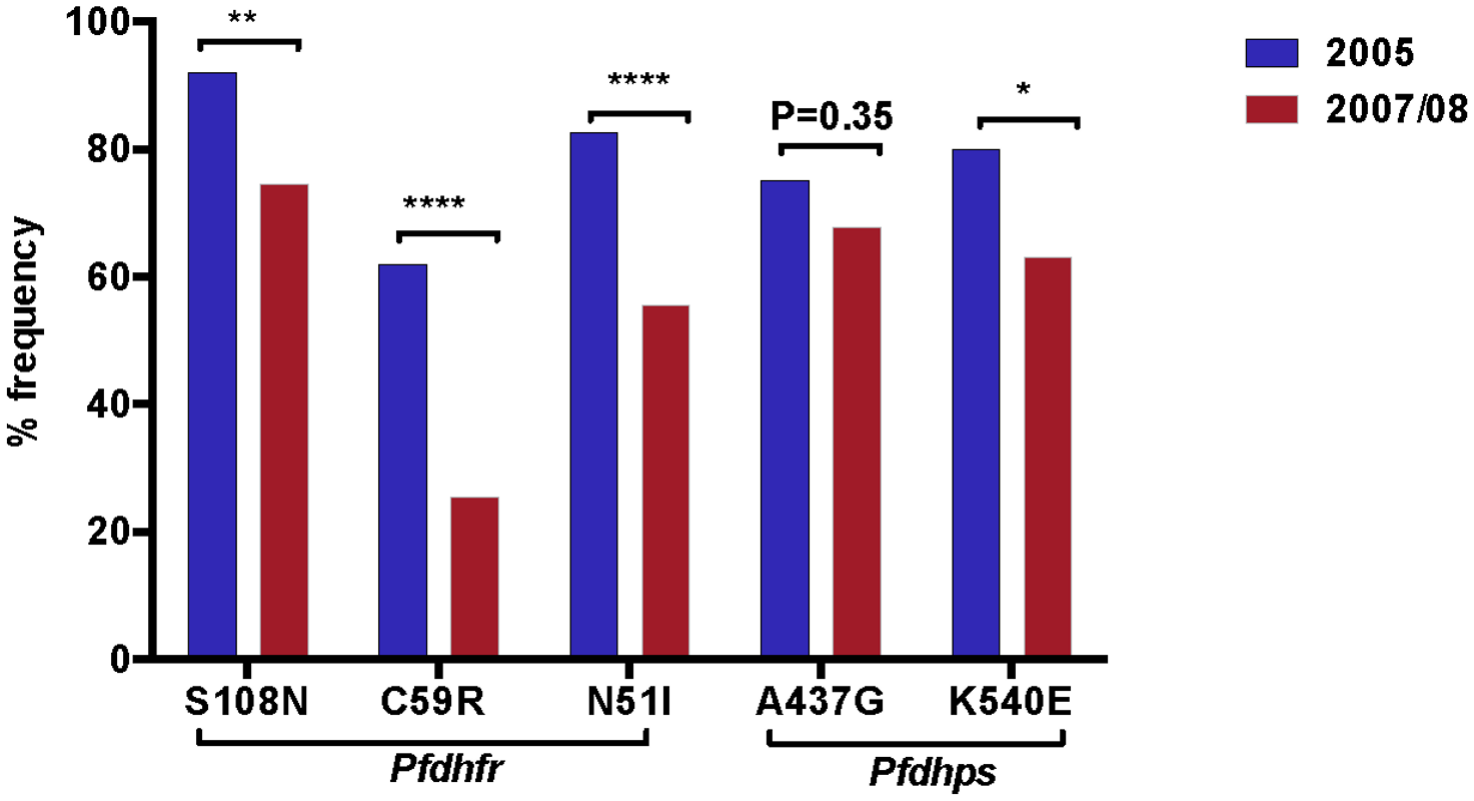
Temporal changes in the frequency of *Pfdhfr* and *Pfdhps* single nucleotide mutations between 2005 and 2007/08. The asterisk indicates the statistical significance of the difference according to Fisher’s exact test.

**Fig 3:**
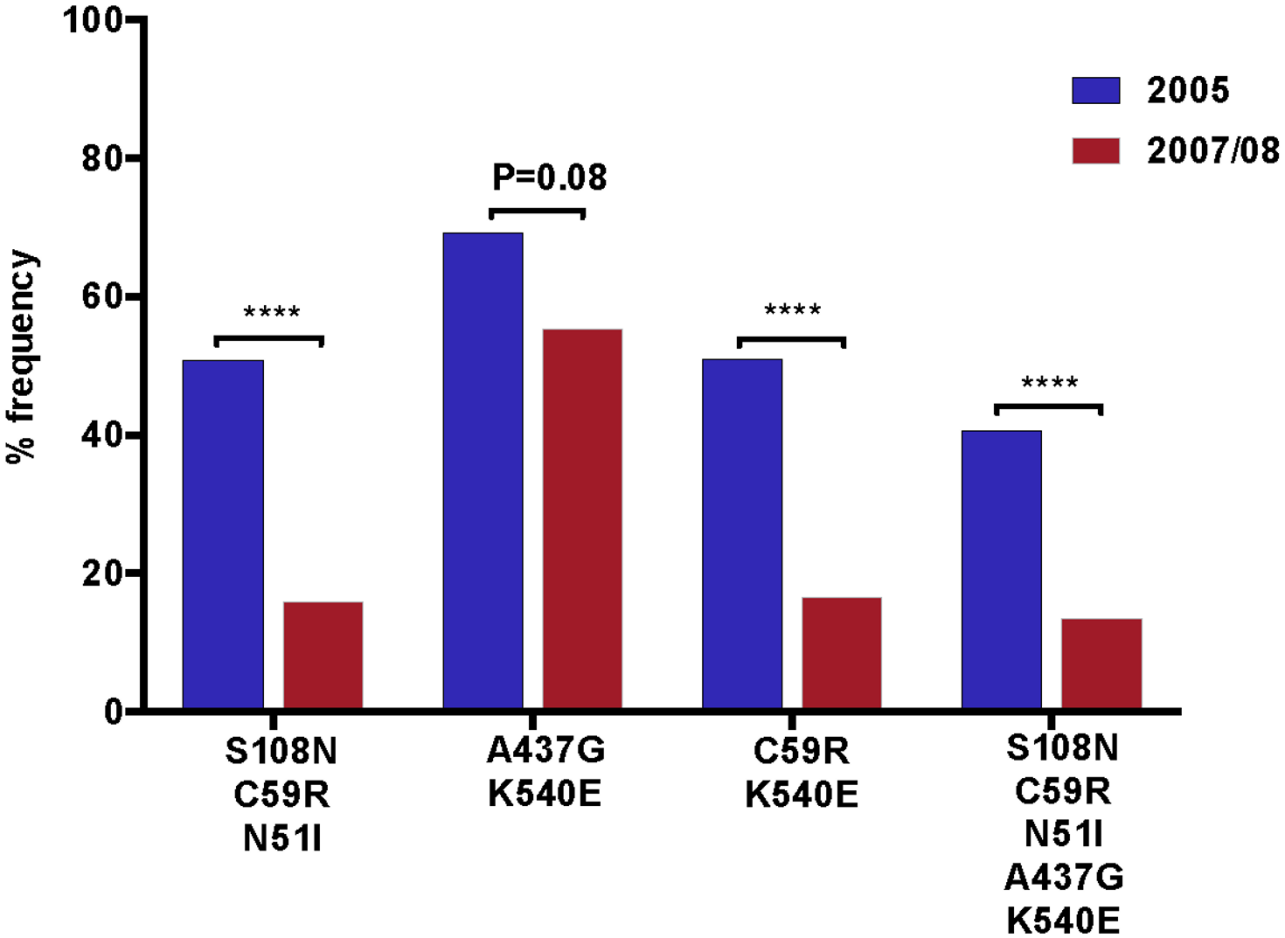
Temporal changes in the frequency of *Pfdhfr* and *Pfdhps* combined mutations. The percentage frequencies of *Pfdhfr* triple (S108N/C59R/N51I), *Pfdhps* double (A437G/K540E) and quintuple mutations (S108N/C59R/N51I/A437G/K540E) were compared between 2005 and 2007/08. The asterisk indicates the statistical significance of the difference according to Fisher’s exact test.

The frequency of the *Pfdhfr* S108N mutation decreased from 92% (58/63) in 2005 to 74.6% (47/63) (P = 0.002) in 2007/08. The relative frequencies of the *Pfdhfr* C59R and N51I mutations decreased from 62% (39/63) to 25.4% (16/63) (P<0.0001) and from 82.5% (52/63) to 55.5% (35/63) (P <0.0001) in 2005 and 2007/08, respectively. *Pfdhps* mutation at position 437 decreased from 75.4% (49/65) to 69.8% (44/65) (P = 0.35) and at position 540 decreased from 80% (52/65) to 63% (41/65) in 2005 and 2007 (P = 0.012), respectively (Fig 2). Change in the allelic frequencies of the *Pfdhfr* triple (S108N/C59R/N51I), *Pfdhps* double (A437G/K540E) and quintuple (the three *Pfdhfr* and two *Pfdhps)* mutations mirrored the trends observed in the prevalence analyses. The frequency of parasites carrying quintuple mutations decreased from 40.7% (24/59) to 13.6% (8/59) (P<0.0001) between 2005 and 2007/08. A significant decrease in the frequency of *Pfdhfr* triple mutation (S108N/C59R/N51I), from 50.8% (32/63) to 15.87% (10/63) (P<0.001), was detected. For *Pfdhps* double mutation (A437G/K540E), there was only a marginal difference between the two surveys with a frequency 69.2% (45/65) and 55.4% (40/65) (P = 0.08) in 2005 and 2007/08, respectively (Fig 3).

### Temporal changes in *Pfdhfr* and *Pfdhps* wild types

**Fig 4:**
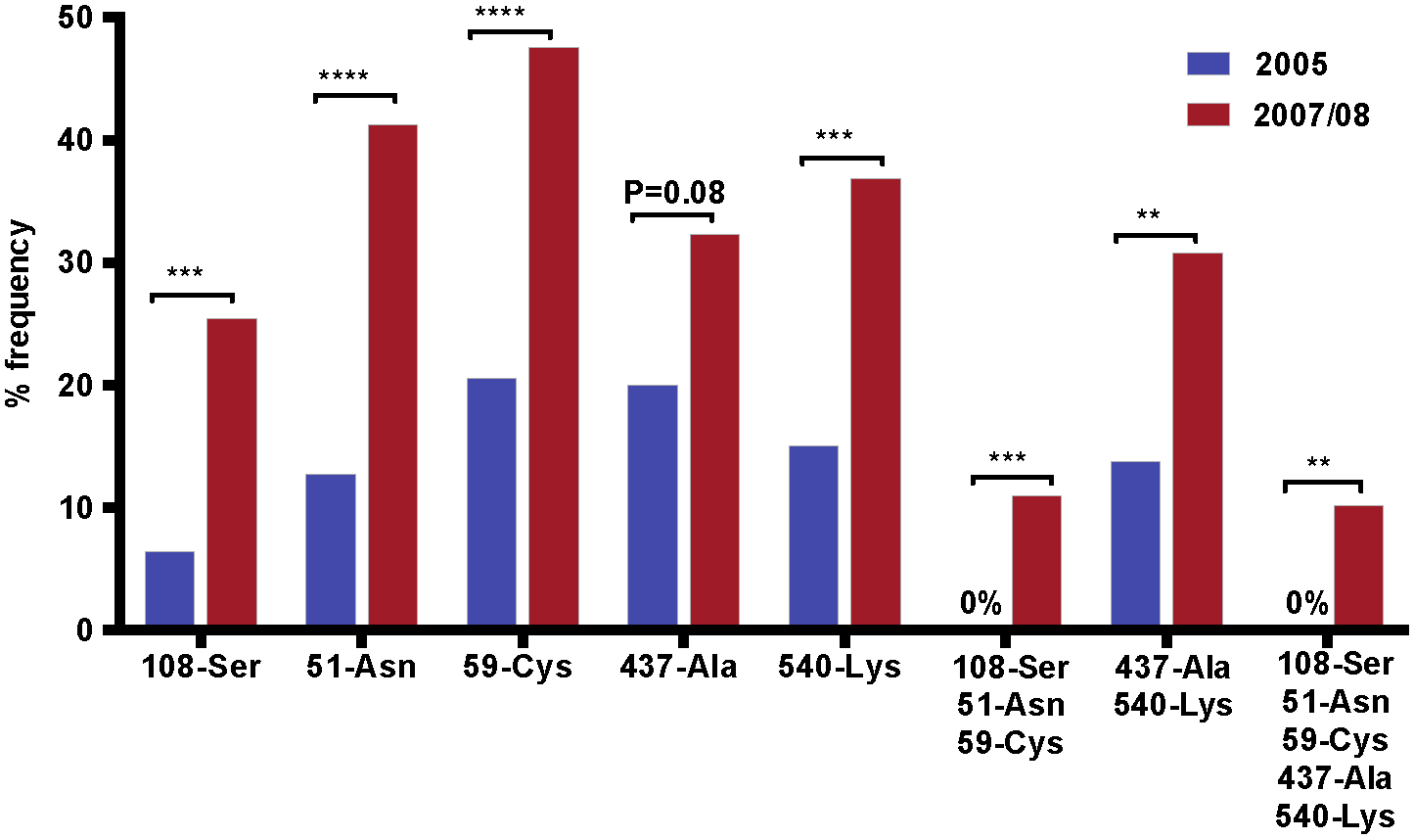
Change in the frequency of *Pfdhfr* and *Pfdhps* wild type alleles. The percentage frequency of wild type alleles in *Pfdhfr* single and triple (Ser-108/Asn-51/Cys-59), *Pfdhps* single and double (Ala-437/Lys-540) and quintuple wild types (Ser-108/Asn-51/Cys-59/Ala-437/Lys-540) were compared between 2005 and 2007/08. The asterisk indicates the statistical significance of the difference according to Fisher’s exact test.

A significant increase in the frequency of *Pfdhfr* and *Pfdhps* wild type was observed in 2007/08 as compared to 2005. Wild type allele in *Pfdhfr* codon 108-Ser increased from 6.35% (4/63) to 25.4% (16/63) (P = 0.0005), codon 51-Asn from 12.7%(8/63) to 41.3% (26/63) (P<0.0001), and codon 59-Cys from 20.63% (13/63) to 47.62% (30/63) (P<0.0001). For *Pfdhps* 437-Ala marginally increased from 20% (13/65) to 32.3% (21/65) (P = 0.08) and 540-Lys wild type allele significantly increased from 15% (10/65) to 36.9% (24/65) (P = 0.0006) (Fig 4). Furthermore, triple *Pfdhfr* wild types were not detected in 2005 but we found the triple wild type *Pfdhfr* in 11.11% (7/63) (P = 0.0007) of the isolates in 2007/08. *Pfdhps* double wild types increased from 13.8% (9/65) to 30.8% (20/65) (P = 0.0063). The *Pfdhfr/Pfdhps* quintuple wild type increased significantly from zero to 10.2% (P = 0.0015), after the withdrawal of SP in 2004 (Fig 4).

## Discussion

There are widely differing malaria endemicity and transmission regions in Ethiopia ranging from a sporadic cases in highland fringe areas to a perennial transmission south western lowlands [2]. Considering the substantial burden of malaria in this country, in 2004 multi-site survey demonstrated mean SP treatment failure rates of 36% and 72% within 14 and 28 days of follow-up, respectively [1]. Following this report, SP was replaced with artemisinin-based combination therapy, artemether/lumefantrine (AL). Different studies show that CQ withdrawal from use for a number of years has reversed resistance based on prevalence of *Pfcrt* resistance marker [19]. This was only possible because CQ was totally banned and it was not available to health facilities. Similarly, SP was banned in Ethiopia since 2004 and unlike many other African countries it has not been used for intermittent preventive treatment. This gives us the opportunity to study the temporal changes in the frequency of molecular markers associated with sulfadoxine/pyrimethamine resistance in the absence of drug pressure in a perennial malaria transmission setting. We compared 159 samples collected in two time points (2005 and 2007/8) by PCR based dot blot hybridization technique. We found a significant declining trend of Sulfadoxine/pyrimethamine mutation markers and re-emergence of wild parasites after the withdrawal of the drug in 2004.

The quintuple mutation (*Pfdhfr* S108N/N51I/C59R/*Pfdhps* A437G/K540E), which was strongly correlated to SP treatment failure of *P*. *falciparum* malaria in south eastern Africa [13], was decreased significantly from 40.7% in 2005 to 13.6% in 2007/08. The level of mutation is much higher as compared to previous report of 1.4% prevalence in the absence of SP in the Ashanti Region of Ghana [36]. The quintuple predictor [13], *Pfdhfr* C59R and *Pfdhps* K540E double mutant, showed a statistically significant change from 51% in 2005 to 16.5% in 2007/08 (P<0.0001) supporting the hypothesis that the resistant strains are less fit in the absence of drug pressure.

Triple mutations in the *Pfdhfr* gene are associated with 60 to 70% rates of SP treatment failure [13]. The *Pfdhfr* triple mutations decreased from 51% to 16% (P<0.001) and the *Pfdhps* double mutation decreased from 69% and 55.4% (P = 0.08) indicating that *Pfdhfr* allele with triple mutations is being replaced faster than the *Pfdhps* allele with double mutations as the drug pressure was removed from the population, suggesting that pyrimethamine sensitivity is reemerging faster than sulfadoxine sensitivity. This is also supported by the increase in the triple wild *Pfdhfr* types from zero to 11.1% (P<0.0001) and *Pfdhps* double wild types from 13.8% to 30.8% (P = 0.006) in the two time points. These findings contrasts with the report from Peru, which showed a decrease in *Pfdhps* 437/540/581 mutation from 47% in 1997 to zero in 2005/6 while *Pfdhfr* triple mutation was decreased to 17% [37]. However, the transmission of malaria in this part of Ethiopia is higher than Peru and the time difference in the Peruvian study was five years. This indicates assessing the change in frequency of mutations in larger time points will give better resolution depending on the intensity of transmission. A similar study in southern Mozambique between 1999 and 2004 showed that the frequency of *dhfr* triple mutation increased from 1999 to 2004, however, the frequency of *dhps* double mutation increased to 2001 but declined to baseline levels by 2004. Quintuple mutations increased from 1999 to 2001 but decreased by 2004, which corresponded with replacement of SP with artemether—lumefantrine as malaria treatment policy in neighboring KwaZulu-Natal, South Africa [28]. Similarly, in Tanzania a community-based study compared the prevalence of mutation between IPTi intervention and comparison groups showed an increase in *Pfdhfr* triple mutation and stabilized frequencies of *Pfdhps* double mutant parasites following the withdrawal of SP as first-line treatment [27].

The slower decline in prevalence of mutations in *Pfdhps* (Figs 2 and 3) suggests that these mutations may be less deleterious to parasite fitness than are *Pfdhfr* mutations in this study area. Although the precise relationship between mutations in the *Pfdhfr* and *Pfdhps* genes in clinical sulfadoxine/pyrimethamine resistance is unclear, previous data showed that the presence of a sensitive *Pfdhfr* allele is highly predictive of sulfadoxine/pyrimethamine treatment success irrespective of the *Pfdhps* allele [38].

We found a significant decrease in all single mutations except *Pfdhps* A437G (from 75.4% to 69.8%, P = 0.35). These findings contradicts with a recent report from Bahir Dar, which is 230km away from this study area [26]. The prevalence of high level of single and combination mutations in *Pfdhfr* and *Pfdhps* alleles in 2005 is consistent with what was known about SP efficacy in this area during 2003. A mean treatment failure of 32.4% on the 14 days and 74.3% in the 28 days follow-up was reported before SP was withdrawn in 2003 [1]. Interestingly, SP-sensitive *P*. *falciparum* parasites have significantly reemerged after the withdrawal of the drug (Fig 3). Triple *Pfdhfr* wild alleles (Ser-108/Asn-51/Cys-59) increased from zero to 11.1%, which is in contrast with Ghana [36], a decrease from 13.3% in 2001 to 11.9% in 2003 in the absence of SP was reported. Double *Pfdhps* wild forms (Ala-437/Lys-540) increased from 13.8% to 30.8% and the most important quintuple *Pfdhfr/Pfdhps* wild forms increased from zero in 2005 to 10.2% in 2007/08, this finding is in accordance with previous report [23] and shows the reemergence SP sensitive parasites. These finding is also in contradiction with the reported changes in Bahir Dar [26].

The decrease in mutation and subsequent increase wild type parasites might be explained by the reduction of residual drug-resistant parasites, caused by the strong drug pressure imposed before 2004 when SP was the first-line drug, followed by lower fitness of these resistant parasites in the absence of drug pressure. Re-emergence of SP susceptible parasites support the hypothesis that drug-resistant *P*. *falciparum* parasite may be at competitive disadvantage when drug pressure is removed in agreement with epidemiological reports from northern Ethiopia [26], Tanzania [27], Southern Mozambique [28] and Peru for SP [37] and Malawi [19], Sudan [39] and Southeast Asia [40,41] for CQ. However, in Ghana, Cambodia and Venezuela SP resistance alleles have remained at a high frequency after the replacement of SP [24,25,36].

Although the change in the frequency of mutation is significant, the resistant alleles are still abundant in the study area which is explained by the following factors: the time difference between the two surveys was only two and half years and after the 2004 discontinuation of SP for the treatment of *P*. *falciparum* malaria, availability of AL was limited and 85% of the populations were living in rural areas with restricted access to health care giving rise to a high rate of presumptive treatment with available drugs like SP and CQ [42]. The other important factor is cross-resistance between SP and Trimethoprim/ sulfamethoxazole [43,44]. The uses of Trimethoprim/ sulfamethoxazole as prophylaxis against human immunodeficiency virus (HIV) associated opportunistic infections most likely make an essential contribution to SP resistance. In this study, we do not have the data for the HIV prevalence or positivity of the study populations and we cannot sort out the impact of trimethoprim/sulfamethoxazole in maintaining the *Pfdhfr* and *Pfdhps* mutation. Cross-resistance between SP and Trimethoprim/sulfamethoxazole appears to be a contributing factor rather than the exclusive factor responsible for less rapid decrease of resistant parasites [43].

The declining trend of mutant alleles and re-emergence of SP sensitive parasites in this perennial transmission area is encouraging despite the limitations of this study. First, this study did not include the change in the frequency of *Pfdhps* A581G mutation in this study area. However, different studies showed the association of A581G mutation and SP resistance in this part of Africa. Second, the methodologies used in this study detect the mutant and wild type alleles at each locus independent of the other. Sequencing of the full-length *Pfdhfr* and *Pfdhps* genes will give a better resolution to determine combinations of mutations. Finally, this study reports the change in frequency of mutation only with two time points, which are 2.5 years apart. Large scale, multi-centered studies with multiple time points will be required to investigate the change in the frequency of mutation and re-emergence of SP sensitive parasites in different transmission areas of the country.

The recovery of SP-sensitive parasite populations in the study area after removal of drug pressure suggests an advantage of the native *Pfdhfr* and *Pfdhps* molecule over its mutant forms and points to the possible value of drug rotation strategies in antimalarial policies as we facing the advent of artemisinin resistance. The use of SP for the control and elimination of malaria together with the current tools will require much lower rates of mutation associated with SP resistance. Further multi-centered studies are required to monitor the change in the frequency of mutation, in order to monitor and prevent the establishment of multi drug resistant parasites in different parts of the country.

## Supporting Information

S1 Table Primer Sequences and PCR conditions for the amplification reaction. (DOCX)

S2 Table List of positive controls used in the dot blot hybridization experiment. (DOCX)

S1 Dataset *Pfdhfr* and *Pfdhps* wild and mutant type raw data for 2005 and 2008. (XLSX)
