# Supplementary material for: Declining trend of Plasmodium falciparum dihydrofolate reductase (dhfr) and dihydropteroate synthase (dhps) mutant alleles after the withdrawal of Sulfadoxine-Pyrimethamine in North Western Ethiopia
Source: PLoS One. 2015 Oct 2;10(10):e0126943. doi: 10.1371/journal.pone.0126943 (PMC4591967; doi:10.1371/journal.pone.0126943)
Supplement: S1 Table — (DOCX) [file pone.0126943.s001.docx]

**S1 Table. Primer Sequences and PCR conditions for the amplification reaction**

| PCR type | Gene | Primers Names and Sequences  (MWG Biotech, Germany) | Cycling Conditions |
| --- | --- | --- | --- |
| Primary PCR | *Pfdhfr* | AMP1 forward: TTTATATTTTCTCCTTTTTA  AMP2 reverse: CATTTTATTATTCGTTTTCT | 95^o^C for 3min  92^o^C for 30 sec; 45oC for 45 sec and 72^o^C for 45 sec for 45 cycles and a final cycle of 72^0^C for 5 min |
|  | *Pfdhps* | 186 forward: GTTTAATCACATGTTTGCACTTTC  M3717 reverse:  CCATTCCTCATGTGTATACACAC | 95^o^C for 3min  92^o^C for 30 sec; 50oC for 45 sec and 72^o^C for 60 sec for 30 cycles and a final cycle of 72^0^C for 5 min |
| Nested PCR | *Pfdhfr* | SP1 forward:  ATGATGGAACAAGTCTGCGAC  SP2 reverse:  ACATTTTATTCGTTTTC | 95^o^C for 3min  92^o^C for 30 sec; 45oC for 30 sec and 72^o^C for 30 sec for 30 cycles and a final cycle of 72^0^C for 5 min |
|  | *Pfdhps* | 218 forward: TAATAGCTGTAGGAAGCAATTG  185 reverse:  TGATACCCGAATATAAGCATAATG | 95^o^C for 3min  92^o^C for 30 sec; 48oC for 30 sec and 72^o^C for 30 sec for 30 cycles  and a final cycle of 72^0^C for 5 min |
