# Supplementary material for: Declining trend of Plasmodium falciparum dihydrofolate reductase (dhfr) and dihydropteroate synthase (dhps) mutant alleles after the withdrawal of Sulfadoxine-Pyrimethamine in North Western Ethiopia
Source: PLoS One. 2015 Oct 2;10(10):e0126943. doi: 10.1371/journal.pone.0126943 (PMC4591967; doi:10.1371/journal.pone.0126943)
Supplement: S2 Table — (DOCX) [file pone.0126943.s002.docx]

## S2 Table. List of positive controls used in the dot blot hybridization experiment. The highlighted clones were used as a positive control for each wild and mutant probes. Mutant amino acids are indicated in bold.

| Clone  (Genomic DNA) | *Pfdhfr* | | | | | | *Pfdhps* | | | |
| --- | --- | --- | --- | --- | --- | --- | --- | --- | --- | --- |
|  | 51 | | 59 | | 108 | | 437 | | 540 | |
|  | Asn | Ile | Cys | Arg | Ser | Asn | Ser | Gly | Lys | Glu |
| 3D7 |  |  |  |  |  |  |  |  |  |  |
| Dd2 |  |  |  |  |  |  |  |  |  |  |
| T994 |  |  |  |  |  |  |  |  |  |  |
| HB3 |  |  |  |  |  |  |  |  |  |  |
| T996 |  |  |  |  |  |  |  |  |  |  |
| SL/D6 |  |  |  |  |  |  |  |  |  |  |
| IEC513/86 |  |  |  |  |  |  |  |  |  |  |
